# Supplementary material for: Speed-dependent changes in the arm swing during independent walking in individuals after stroke
Source: PLoS One. 2025 Jan 3;20(1):e0315332. doi: 10.1371/journal.pone.0315332 (PMC11698429; doi:10.1371/journal.pone.0315332)
Supplement: S1 File — (DOCX) [file pone.0315332.s001.docx]

Supporting information

**S1** **Mean different curves of significant differences detected between both conditions.**

1. **Full group**

**
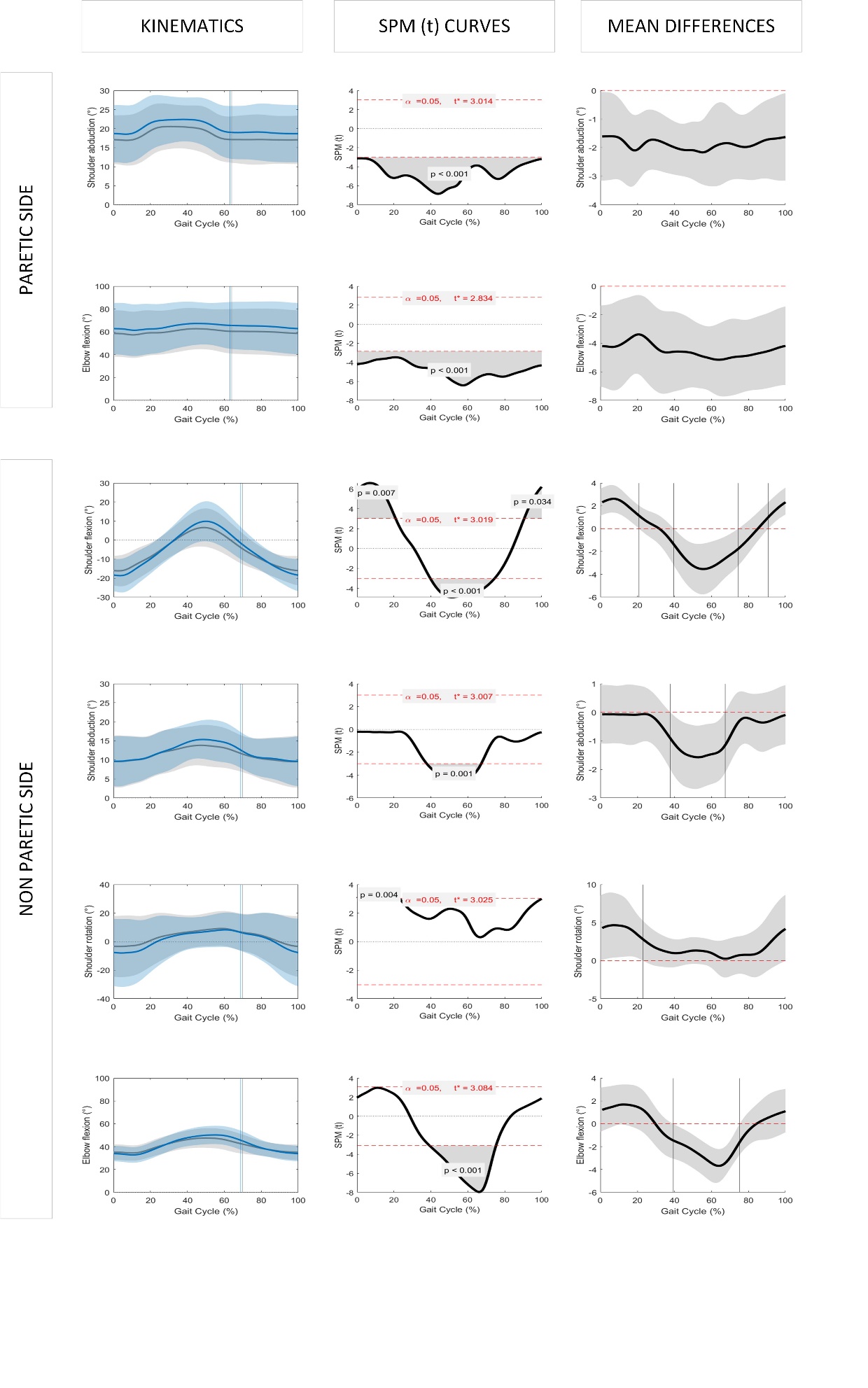
**

1. **1:1 group**

**
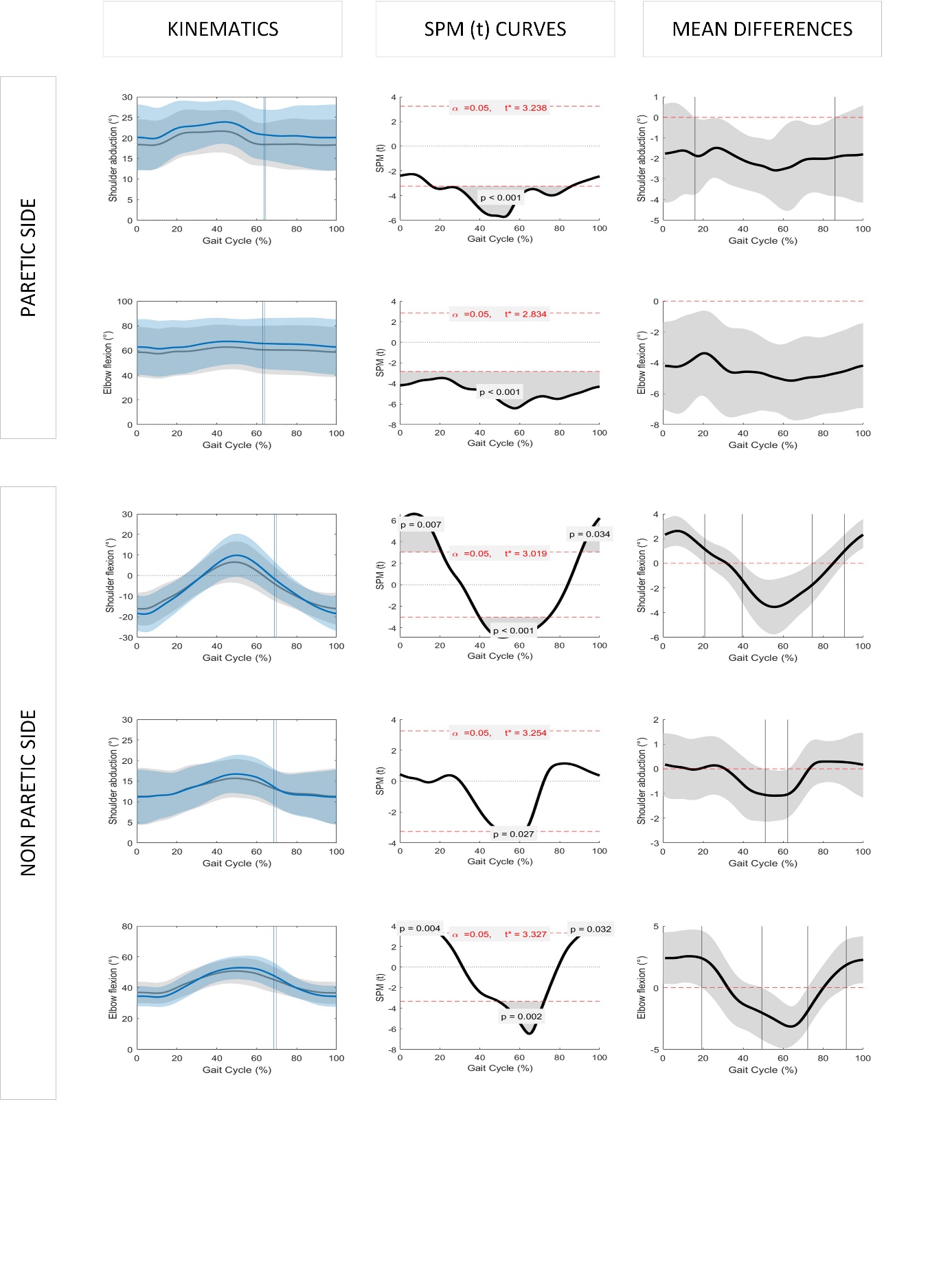
**

1. **2:1 group**

**
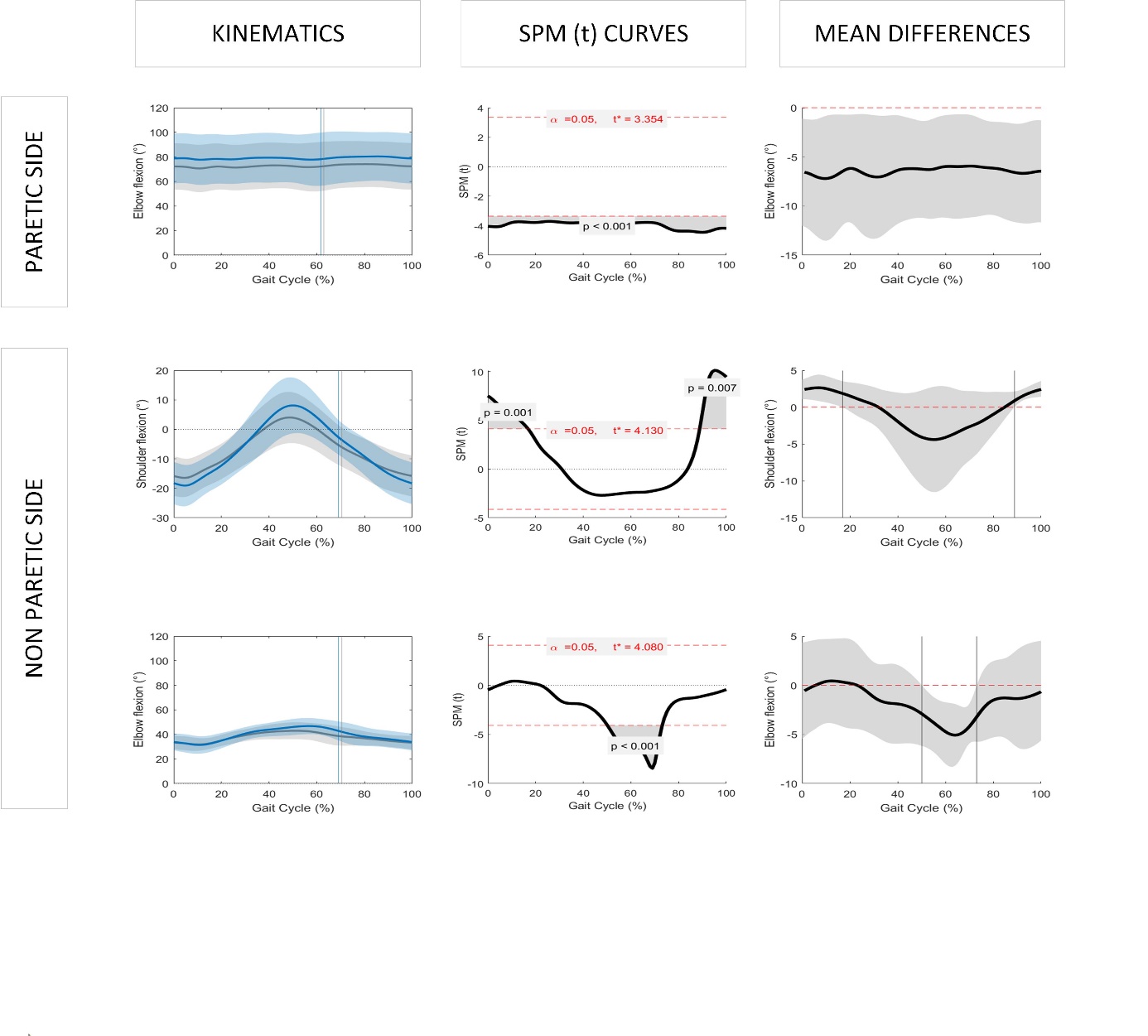
**

The left column shows the mean kinematic curves and standard deviations for the CWS condition (grey) and FWS condition (blue). The vertical lines indicate the moment of push-off in each condition. The middle column displays the SPM (t) curves and indicates where the critical threshold was exceeded (t*). The right column displays the mean difference curves. The vertical lines indicate the start of end point of statistical significance as indicated in the middle column.

**S2** **Kinematic curves of the trunk in for all groups.**

***
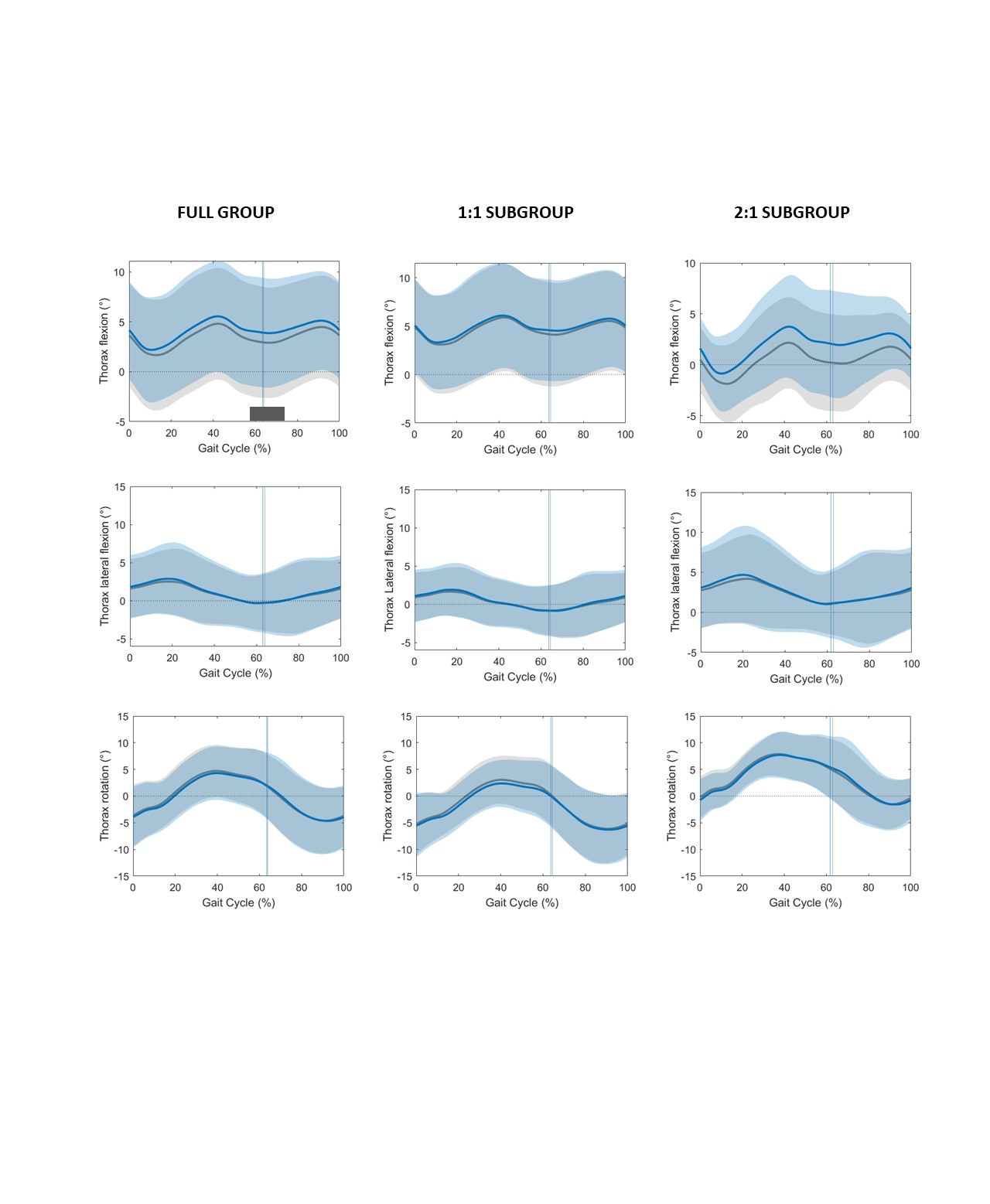
***

The grey lines represent the average kinematic curves and standard deviations of the CWS condition, the blue lines represent the average kinematic curves and standard deviations of the FWS condition. Parts of the gait cycle where significant differences were detected are indicated by dark grey bars. The vertical lines indicate the moment of push-off in each condition.

**S3** **Visual representation of the paretic shoulder range of motion in the sagittal plane (°) for the two conditions for each participant.**

**S4** **Individual graphs for the paretic shoulder movement in the sagittal plane (°) displaying the heterogeneity in timing of maximal shoulder flexion.**
