# Supplementary material for: Speed-dependent changes in the arm swing during independent walking in individuals after stroke
Source: PLoS One. 2025 Jan 3;20(1):e0315332. doi: 10.1371/journal.pone.0315332 (PMC11698429; doi:10.1371/journal.pone.0315332)
Supplement: Attachment — Submitted filename: PONE-D-24-22190 Review.docx [file pone.0315332.s003.docx]

Date:	9/25/2024

Subject:	Reviewer Comments

“Speed-dependent changes in the arm swing during independent walking in individuals after stroke.”

Manuscript ID: PONE-D-24-22190

**Overall Summary**

The purpose of this study was to describe differences in upper extremity kinematics in response to speed changes for persons with stroke. A secondary purpose was to test for differences in subgroups based on 1:1 or 2:1 arm swing pattern.

In general, the authors used appropriate research design, analytical methods, and statistical analyses. The manuscript was clearly written, and the figures presented data visualization well. I have some comments for the authors to consider.

**Major Concerns**

1. The authors claim that there is a causal relationship between walking speed and upper extremity movements to support rationale for treatment. For example see line 96. However, the authors do cite supporting evidence to demonstrate a causal relationship, nor does the current study design provide evidence for causality as it is a descriptive, cross-sectional design. I would caution the authors about using statements that suggest causality in the introduction and conclusion.
2. The authors do not discuss their findings with respect to passive mechanical coupling that can occur as a result of increased speed. For example, increases in upper extremity ROM can be the result of mechanical transfer of ground reaction forces through the skeletal, ligamentous, and passive properties of muscle tissue rather than active muscle contractile properties. Adding this possible hypothesis to the discussion will provide a more comprehensive picture for readers. Additionally, the current study design does not differentiate whether the observed changes are a result of impairment or an artifact of increased speed. The use of the term speed-dependency would imply that the resultant changes are not influenced by impairment. Further studies will be needed to elucidate these differences.
3. The discussion could be strengthened by addressing explicitly whether the findings supported or refuted the proposed hypotheses.

**Minor Concerns**

Abstract:

Line 49. This sentence appears to be incomplete?

Introduction:

Line 103-105. The introduction would be strengthened for readers if these subgroup patterns were introduced prior to the study purpose.

Results:

Line 250-254. Please consider re-wording this section to clarify if these are descriptive characteristics of the group or relationships between variables within the subgroups. I had to return to tables to try and understand the clinical descriptives for each subgroup.

Discussion:

Line 276-277. Please consider deleting as this statement does not describe how the findings support or refute the hypotheses.

Limitations:

Please add that these results may not be generalizable to all persons post-stroke provided the high functional level based on gait speed.
